# Changes in the Proportion of Gastrointestinal Emergency Endoscopy and Peptic Ulcer Disease During the COVID-19 Pandemic: A Local Retrospective Observational Study From Vietnam

**DOI:** 10.3389/fpubh.2022.699321

**Published:** 2022-02-18

**Authors:** Hang Viet Dao, Long Bao Hoang, Nha Ngoc Hoa Le, Trang Thi Thu Tran, Hung Manh Nguyen, Long Van Dao, Ngoan Tran Le

**Affiliations:** ^1^Internal Medicine Department, Hanoi Medical University, Hanoi, Vietnam; ^2^Research and Training Management Department, Institute of Gastroenterology and Hepatology, Hanoi, Vietnam; ^3^Gastroenterology Division, Internal Medicine and Hematology Department, Gastrointestinal Endoscopic Center, Semmelweis University, Budapest, Hungary; ^4^Hanoi University of Pharmacy, Hanoi, Vietnam; ^5^Institute of Research and Development, Duy Tan University, Da Nang, Vietnam; ^6^Department of Public Health, International University of Health and Welfare, Otawara, Japan

**Keywords:** COVID-19, peptic ulcer disease, upper gastrointestinal endoscopy, endoscopy volume, emergency endoscopy, healthcare reform, pandemic

## Abstract

**Objectives:**

The coronavirus disease 2019 (COVID-19) pandemic has disrupted the practice of gastrointestinal (GI) endoscopy units and may increase the risk of digestive disorders. We described the situational changes in GI endoscopy and peptic ulcer disease (PUD) proportion during COVID-19 in Vietnam and examined the associated factors.

**Methods:**

A retrospective ecological study was conducted on data of Hanoi Medical University Hospital, Vietnam. The number of upper GI endoscopy and the proportion of GI emergency endoscopy and PUD were compared between 2019 and 2020 by month (January to June). Log-binomial regression was used to explore associated factors of GI emergency endoscopy and PUD.

**Results:**

The number of endoscopies decreased remarkably during the nationwide social distancing in April 2020. Compared to April 2019, the proportion in April 2020 of both GI emergency endoscopy [4.1 vs. 9.8%, proportion ratio (PR) 2.39, 95% CI 2, 2.87], and PUD [13.9 vs. 15.8%; PR, 1.14; 95% CI, 1.01, 1.29] was significantly higher. In log-binomial models, the proportion of GI emergency endoscopy was higher in April 2020 compared to April 2019 (adjusted PR, 2.41; 95% CI, 2.01, 2.88). Male sex and age of ≥50 years were associated with an increased PUD and GI emergency conditions.

**Conclusion:**

The proportion of both GI emergency endoscopy and PUD was significantly higher during the time of the state of emergency due to the ongoing COVID-19 pandemic in 2020 when compared to 2019 at the same health facility in Vietnam. The findings suggest that healthcare delivery reforms during the era of an emerging pandemic are required to reduce digestive disorders, in particular, and chronic diseases in general.

## Introduction

Vietnam reported the first coronavirus disease 2019 (COVID-19) case in January 2020; since then, the country has applied strict measures to control the pandemic, including contact tracing, quarantine, isolation, and social distancing (1). Although the first wave of the pandemic was successfully confined, a sharp increase in the number of cases in late March 2020 led to enforcement of nationwide distancing in early April 2020 (1). During this period, Vietnamese hospitals had strongly limited outpatient activities, and doctors also restricted their upper gastrointestinal (GI) endoscopy indications and referrals to certain patient populations. This was partly based on the guideline from the Asian Pacific Society of Digestive Endoscopy (A-PSDE) issued in April 2020, stating that upper GI endoscopy should only be performed in emergency cases (2).

Some studies have reported a delayed diagnosis of GI cancer due to the impact of COVID-19 (3, 4), but the situational change in the diagnosis of peptic ulcer disease (PUD) during COVID-19 has not been widely reported. PUD, a commonly detected condition during upper GI endoscopy, was responsible for more than 300,000 deaths globally in 2013, mostly due to GI hemorrhagic complications (5), and was associated with high mortality despite advances in endoscopic and pharmacological treatment (6, 7). Early detection of PUD contributes to the prevention of GI hemorrhage and diagnosis of gastric cancer.

Social distancing, albeit effective in controlling the pandemic, has been associated with stress and anxiety related to COVID-19 and its consequences (e.g., unemployment and family pressure) (8). During social distancing, people tended to change their dietary habits (9) and engaged in risky behaviors such as smoking and alcohol consumption (10, 11). These factors could increase the incidence of PUD. Also, limited access to healthcare services and minimal indication in upper GI endoscopy during the nationwide distancing could delay PUD diagnosis, which, in our opinion, is also likely to contribute to the increase in complications arising from PUD.

In this study, we described the situation of upper GI endoscopy at Hanoi Medical University Hospital (HMUH), Vietnam, between January and June 2020 and compared it to the same period of 2019. The situation of upper GI endoscopy was described in three domains: volume of upper GI endoscopy, the proportion of GI emergency endoscopy, and proportion of PUD. We hypothesized that there were changes in these domains during the COVID-19 pandemic. We also explore factors that are associated with these changes.

## Materials and Methods

### Study Design

We conducted a retrospective ecological study on the situation of upper GI endoscopy using the data of HMUH. We collected anonymous electronic data of all the outpatients who had an upper GI endoscopy performed between January 1 and June 30 in the years of 2019 and 2020. Data from 2019 were used as a control group because the COVID-19 pandemic has not yet started in early 2019.

Collected data included sex, age, residential address, date of endoscopy, diagnosis on admission, and endoscopic diagnosis. From the date of endoscopy, we obtained the year (2019 or 2020), months (January to June), and week of endoscopy. The week of endoscopy was counted from 1, with the start date of Week 1 being the first Monday before January 2.

We determined whether a patient had GI emergency endoscopy and PUD based on diagnosis on admission and endoscopic diagnosis, respectively. Also, because these data were in the form of free text and were not properly coded, we developed a semi-automated algorithm using the Python programming language to match the keywords in the diagnoses. For the diagnosis of PUD, we looked for gastric ulcer and duodenal ulcer of any region and severity; the set of keywords was created to match all these possible diagnoses. For GI emergency endoscopy, we looked for signs and symptoms that suggested an indication for GI emergency endoscopy for the endoscopies performed during office hours and selected all endoscopies performed out of office hours, during weekends, or on national holidays; the set keywords included hematemesis, melena, foreign body, weight loss, and anemia. The dataset for analysis can be reached at https://doi.org/10.7910/DVN/RMFFHJ on Harvard Dataverse.

During the first wave of the COVID-19 pandemic, the Vietnamese government had started a classification system of COVID-19 risk for cities and provinces since March 31, 2020 (12). A city/province would be classified as low, moderate, or high risk. A high-risk region was a city or province that had active COVID-19 cases or was adjacent to regions that had active COVID-19 cases. Restriction on transportation and social gathering was enforced in these regions (12). Patients from high-risk regions might have had difficulties traveling to HMUH for examination and endoscopy, and this would affect the proportion of GI emergency endoscopy and PUD during the nationwide distancing. Therefore, based on the residential address of the patient, we also classified patients as coming from low-, moderate-, or high-risk regions, and compared the situation of upper GI endoscopy among these regions.

### Sample Size

We estimated the minimum sample size needed to compare the proportion of PUD and emergency endoscopy before and after COVID-19. Sample size calculation was done for emergency endoscopy because the proportion of emergency endoscopy was much lower than that of PUD. Based on our non-published statistics, we assumed a pre-COVID proportion of emergency of 3.5% and a post-COVID proportion of 4%. With a confidence level of 95% and 90% power, the required minimum sample size for each year was 30,737 patients, which was similar to the number of patients in our database.

### Data Analysis

To describe the trend in the number of upper GI endoscopy of patients in relation to the COVID-19 situation in Vietnam, we summarized the weekly number of the patients admitted to HMUH in 2020 and plotted against the weekly number of new COVID-19 cases in Vietnam. The data of new COVID-19 cases were retrieved from the official website of Vietnam's Ministry of Health (13). We also summarized the monthly number of upper GI endoscopies at HMUH to compare between 2019 and 2020; these comparisons were stratified by sex and COVID-19 risk of the patient's residential area.

We calculated the crude monthly proportion of GI emergency endoscopy and PUD, and then stratified by sex and COVID-19 risk of the residential area. The proportion of PUD was also compared between the patients with and without GI emergency endoscopy. All proportions were compared between 2019 and 2020.

Proportion ratios of GI emergency endoscopy and PUD between 2019 and 2020 were estimated by the log-binomial regression instead of binary logistic regression that is adjusted for sex, age group, COVID-19 risk of the residential area, and GI emergency endoscopy (for the model of PUD). We also ran a second model in which we replaced COVID-19 risk of the residential area with a binary variable defined as whether the patients lived in Hanoi. The rationale was that Hanoi was a high-risk region during the outbreak, and thus, travel from other cities/provinces to Hanoi had been limited, which could make the proportion of GI emergency endoscopy and PUD different among the patients who lived in Hanoi and other regions. We constructed six regression models for each month from January to June because we wanted to observe the association in individual months.

Data were processed and analyzed using the Python programming language. The Python packages used in our statistical analyses included *tableone* (14) for making Table 1 and *statsmodels* for the log-binomial model (15).

**Table 1:** Patient characteristics between 2019 and 2020.

	**2019 (*n* = 41,930)**	**2020 (*n* = 30,456)**
Age, mean (SD)	44.0 (15.0)	44.2 (15.1)
**Age group**, ***n*** **(%)**
<18 years	1,753 (4.2)	1,065 (3.5)
18– <30 years	6,432 (15.3)	4,937 (16.2)
30– <40 years	9,983 (23.8)	7,303 (24.0)
40– <50 years	9,110 (21.7)	6,369 (20.9)
50– <60 years	8,615 (20.5)	6,165 (20.2)
Over 60 years	6,037 (14.4)	4,617 (15.2)
Female sex, *n* (%)	23,997 (57.2)	17,084 (56.1)
**COVID-19 risk of residential area**, ***n*** (%)^*^
Low risk	14,254 (34.1)	9,477 (31.2)
Moderate risk	7,375 (17.6)	5,464 (18.0)
High risk	20,214 (48.3)	15,438 (50.8)
Living in Hanoi, *n* (%)^*^	14,617 (34.9)	11,707 (38.4)
**Month**, ***n*** **(%)**
January	5,506 (13.1)	4,525 (14.9)
February	4,924 (11.7)	5,142 (16.9)
March	8,439 (20.1)	3,252 (10.7)
April	6,692 (16.0)	1,867 (6.1)
May	8,252 (19.7)	7,947 (26.1)
June	8,117 (19.4)	7,723 (25.4)
Peptic ulcer disease, *n* (%)	6,223 (14.8)	4,396 (14.4)
Emergency endoscopy, *n* (%)	1,575 (3.8)	1,497 (4.9)

* *Data on residential address were missing in 164 patients*.

### Ethical Consideration

The study was approved by the Institutional Review Board of Hanoi Medical University under Decision No. 130/GCN-HNCYSH-HYHN dated July 22, 2020. All identifiable information in the electronic hospital data had been removed before the dataset was sent to the research team. The research team conducted no attempt to re-identify any patient in the dataset.

## Results

### The Situation in 2020

**Figure 1:**
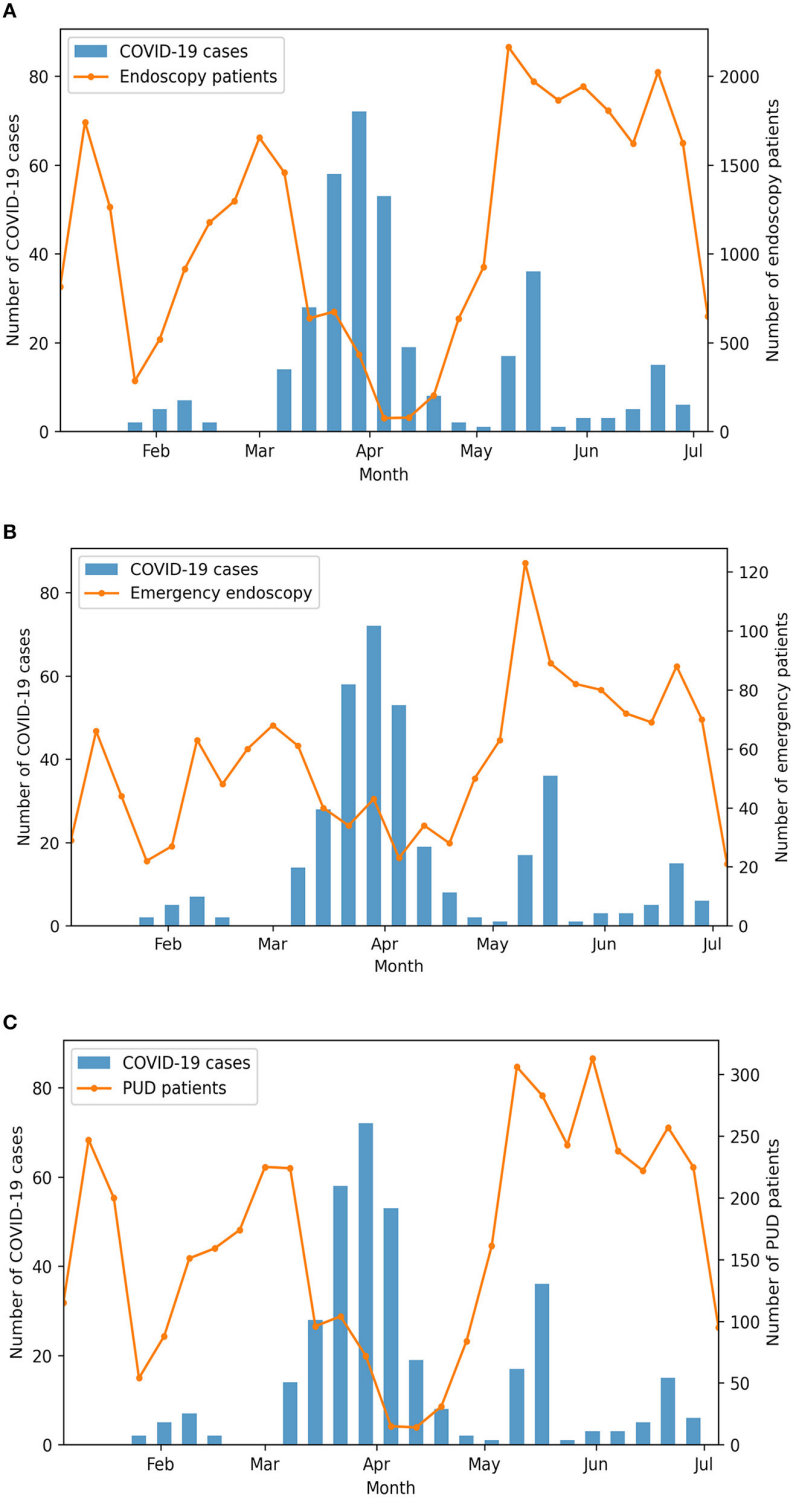
The weekly number of upper gastrointestinal (GI) endoscopy **(A)**, GI emergency endoscopy **(B)**, and peptic ulcer disease (PUD) **(C)** compared against the weekly number of new coronavirus disease 2019 (COVID-19) cases.

Between January 1, 2020 and June 30, 2020, a total of 30,456 patients had upper GI endoscopy performed at the HMUH, among which 1,497 (4.9%) had GI emergency endoscopy and 4,396 (14.4%) had PUD. There were two noticeable drops in the number of the patients (Figures 1A–C). The first drop was in early February; this period is the traditional Tet holiday in Vietnam, when people often avoid visiting the hospitals. The second drop was between mid-March and early May, coinciding with the first wave of COVID-19 in Vietnam. The number of new COVID-19 cases peaked in late March, marking the beginning of the nationwide distancing on April 1.

### Comparison With 2019, Stratified Analysis

The number of patients with upper GI endoscopy in 2020 was remarkably lower than that in 2019 (30,456 in 2020 vs. 41,930 in 2019). The distribution of age, sex, and geographical area was similar between the 2 years (Table 1). The number of patients with upper GI endoscopy dropped remarkably in March and April 2020 compared to the same months in 2019. The overall proportion of PUD was 14.4% in 2020 compared to 14.8% in 2019, and the overall proportion of GI emergency endoscopy was 4.9% in 2020 compared to 3.8% in 2019.

**Figure 2:**
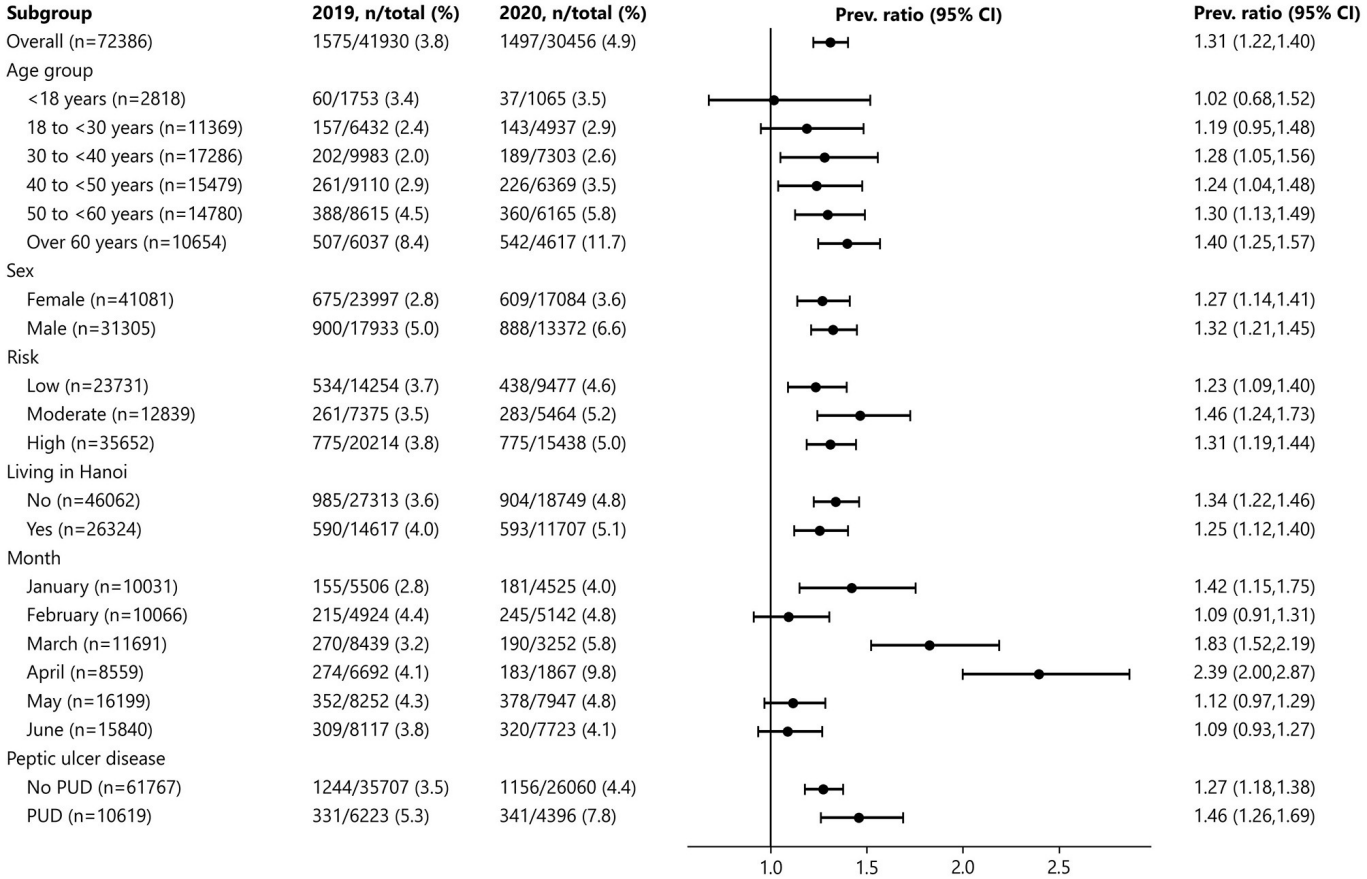
Stratified comparison of the proportion of GI emergency endoscopy between 2019 and 2020.

The proportion of GI emergency endoscopy in 2020 was higher than in 2019 in all subgroups of age, sex, COVID-19 risk of the residential area, and living in Hanoi (Figure 2). The proportion of GI emergency endoscopy in 2020 was significantly lower in February and significantly higher in January, March, and April.

**Figure 3:**
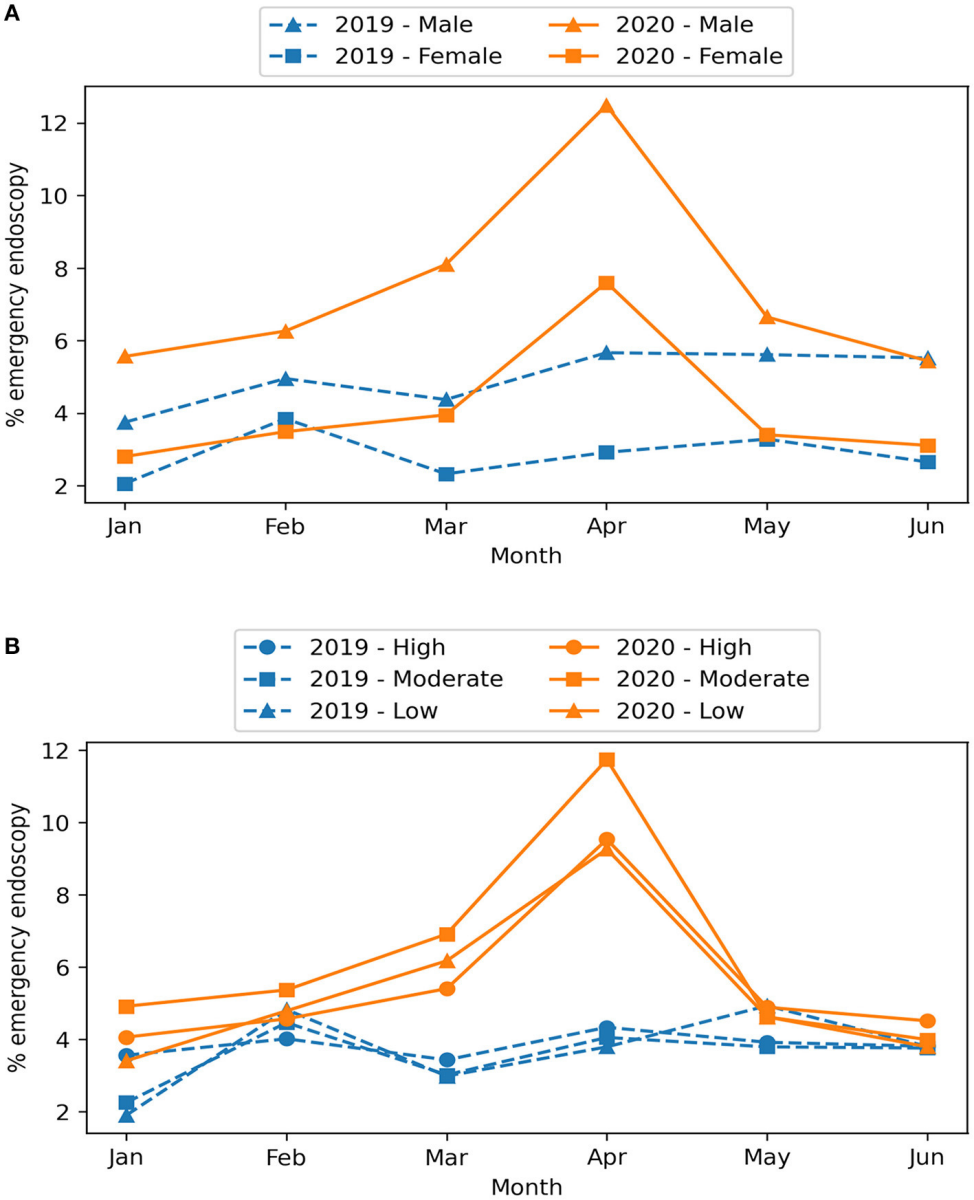
Monthly proportion of GI emergency endoscopy between 2019 and 2020 by sex **(A)** and COVID-19 risk of the residential area **(B)**.

The proportion of GI emergency endoscopy was consistently higher in males than in females and was higher in March and April 2020 (compared to April 2019) in both sexes (Figure 3A). In 2020, the proportion of GI emergency endoscopy in March and April was higher in all three COVID-19 risk regions compared to 2019 (Figure 3B).

**Figure 4:**
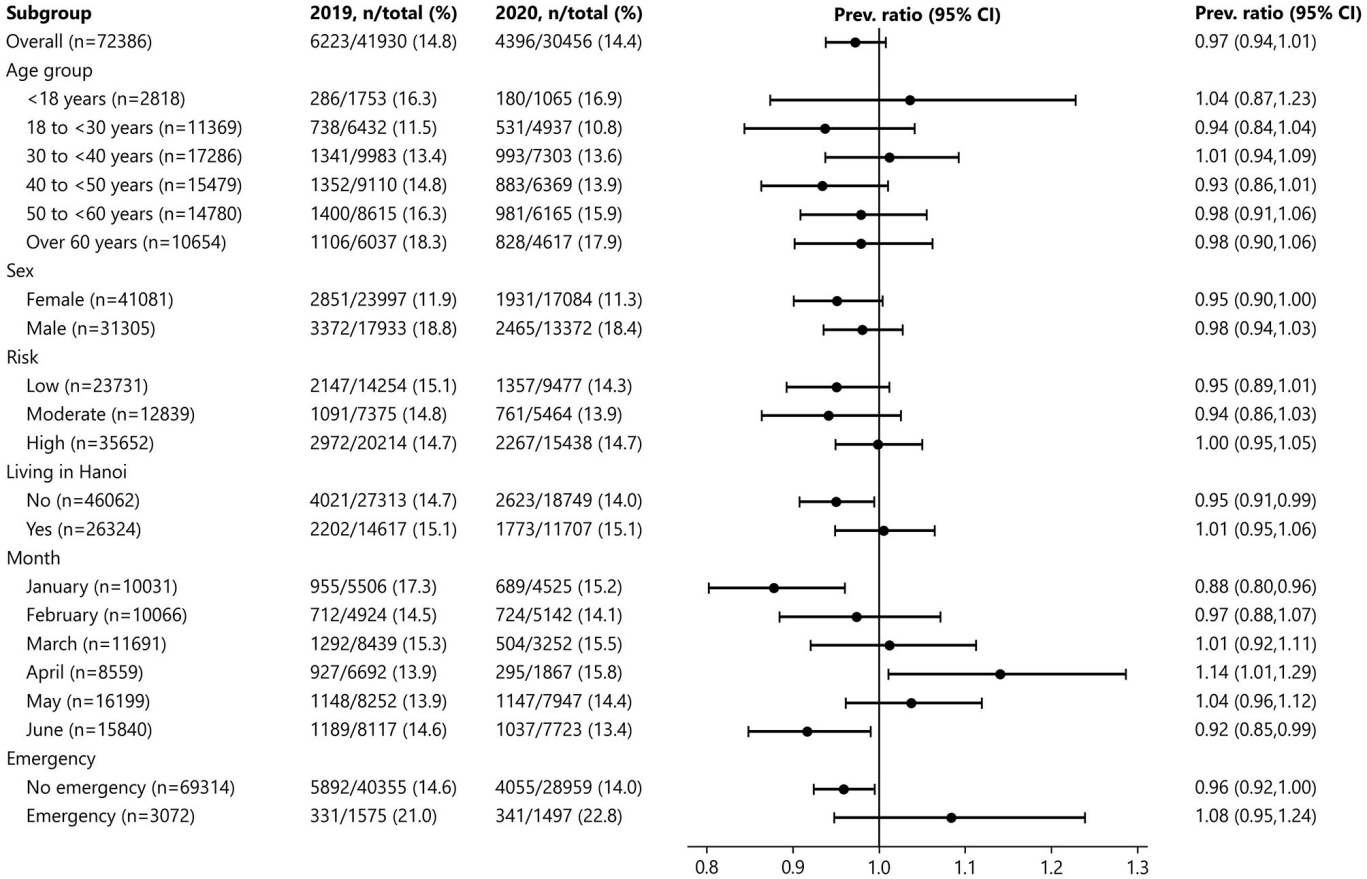
Stratified comparison of the proportion of PUD between 2019 and 2020.

The proportion of PUD between 2019 and 2020 was similar in all subgroups of age, sex, COVID-19 risk of the residential area, living in Hanoi, and GI emergency endoscopy except for a marginally lower proportion in 2020 among the people who did not live in Hanoi and the non-emergency group (Figure 4). While the proportion of PUD in 2020 was significantly lower in January and June, it was significantly higher in April (15.8% in 2020 vs. 13.9% in 2019; PR, 1.14; 95% CI, 1.01, 1.29).

**Figure 5:**
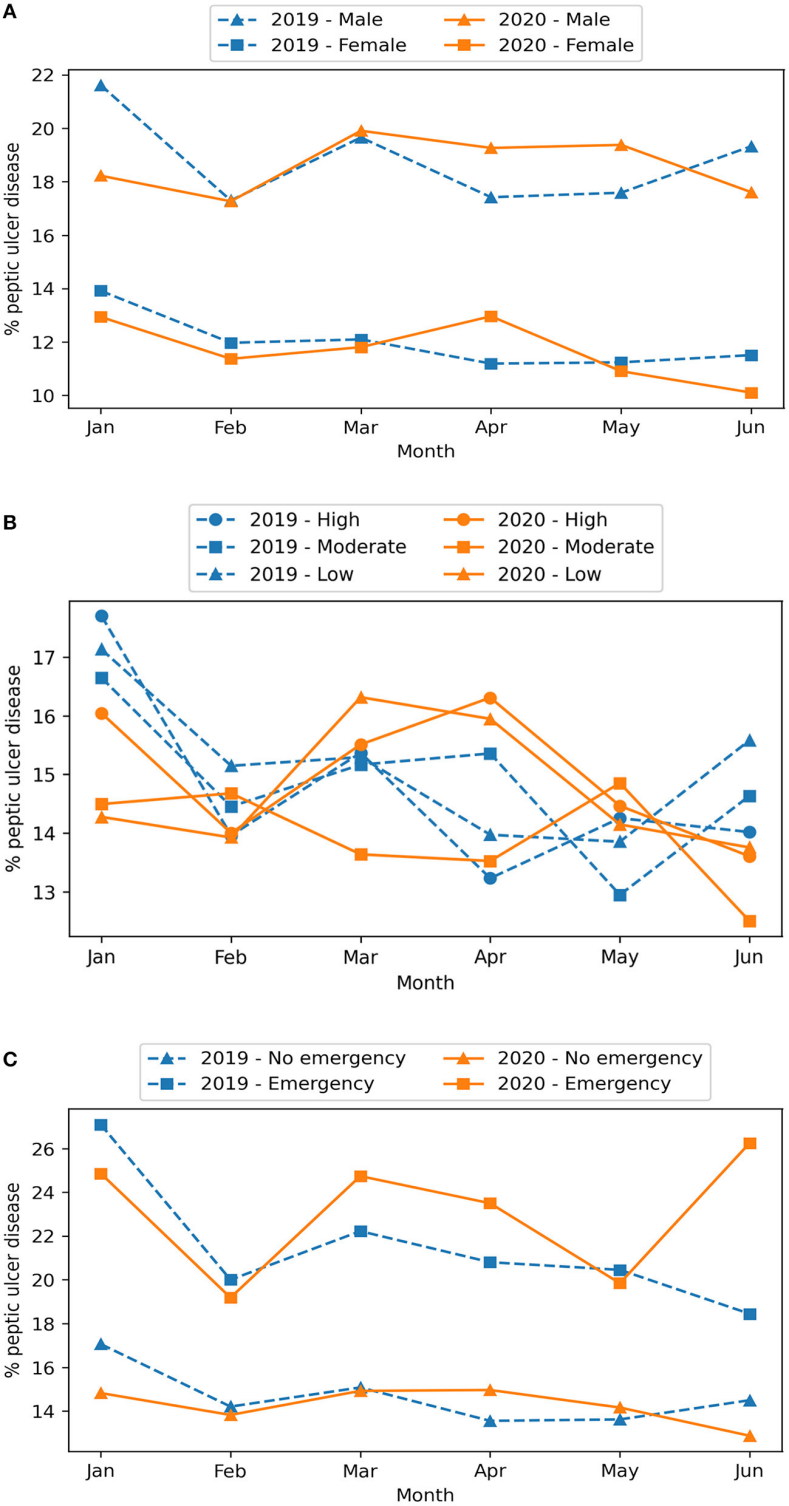
Monthly proportion of PUD between 2019 and 2020 by sex **(A)**, COVID-19 risk of the residential area **(B)**, and GI emergency endoscopy **(C)**.

The proportion of PUD was consistently higher in males than in females and was higher in April 2020 (compared to April 2019) in both sexes (Figure 5A). While the trend in the proportion of PUD in 2019 was similar among the three groups of COVID-19 risk of residential area, the proportion in March and April 2020 appeared to be higher in the high- and low-risk regions compared to 2019, while the moderate-risk region had a lower proportion compared to both the same period in 2019 and the other two risk groups (Figure 5B). In both 2019 and 2020, the proportion of PUD was consistently higher in patients with GI emergency endoscopy (Figure 5C). There was not much difference between 2019 and 2020 in the non-emergency group, but the proportion of PUD was higher in April 2020 in the emergency group.

### Comparison With 2019, Log-Binomial Regression

After adjusting for age, sex, and COVID-19 risk of the residential area, the proportion of GI emergency endoscopy in January, March, and April was significantly higher in 2020 than in 2019 (Tables 2A,B).

**Table 2:** The log-binomial models for the proportion of GI emergency endoscopy.

**Factor**	**January**	**February**	**March**	**April**	**May**	**June**
**A**						
Year 2020	1.42 (1.15, 1.75)	1.07 (0.90, 1.28)	1.85 (1.55, 2.22)	2.35 (1.96, 2.81)	1.11 (0.96, 1.27)	1.02 (0.87, 1.19)
**Age group (reference group: 18–** ** <30 years)**						
<18 years	1.11 (0.55, 2.26)	1.17 (0.61, 2.25)	1.80 (1.00, 3.23)	1.22 (0.70, 2.12)	1.21 (0.74, 1.98)	1.41 (0.90, 2.19)
30– <40 years	0.98 (0.62, 1.54)	0.85 (0.57, 1.27)	1.09 (0.73, 1.62)	0.85 (0.60, 1.20)	0.77 (0.57, 1.05)	0.78 (0.56, 1.09)
40– <50 years	1.15 (0.73, 1.81)	1.67 (1.16, 2.41)	1.65 (1.13, 2.43)	1.12 (0.79, 1.57)	1.27 (0.96, 1.69)	1.05 (0.76, 1.45)
50– <60 years	2.43 (1.61, 3.67)	2.19 (1.54, 3.12)	2.52 (1.76, 3.63)	1.55 (1.12, 2.14)	2.11 (1.62, 2.75)	2.11 (1.58, 2.81)
Over 60 years	4.74 (3.20, 7.01)	5.36 (3.87, 7.43)	4.67 (3.30, 6.61)	2.84 (2.10, 3.83)	3.52 (2.72, 4.55)	3.93 (2.98, 5.18)
Male	2.10 (1.69, 2.60)	1.64 (1.37, 1.96)	2.04 (1.70, 2.44)	1.84 (1.54, 2.20)	1.98 (1.72, 2.29)	2.03 (1.74, 2.37)
**COVID-19 risk of residential area (reference group: high)**						
Low	0.63 (0.49, 0.81)	1.00 (0.82, 1.22)	0.89 (0.72, 1.09)	0.88 (0.72, 1.08)	1.04 (0.89, 1.21)	0.88 (0.74, 1.05)
Moderate	0.81 (0.61, 1.07)	0.97 (0.76, 1.23)	0.90 (0.71, 1.14)	0.96 (0.76, 1.22)	0.87 (0.71, 1.06)	0.88 (0.71, 1.08)
**B**						
Year 2020	1.41 (1.15, 1.74)	1.07 (0.89, 1.27)	1.84 (1.54, 2.21)	2.32 (1.94, 2.78)	1.10 (0.95, 1.26)	1.01 (0.87, 1.17)
**Age group (reference group: 18–** ** <30 years)**						
<18 years	1.13 (0.56, 2.28)	1.18 (0.62, 2.28)	1.80 (1.00, 3.24)	1.22 (0.70, 2.12)	1.21 (0.74, 1.98)	1.42 (0.91, 2.21)
30– <40 years	1.00 (0.64, 1.58)	0.86 (0.57, 1.28)	1.09 (0.73, 1.63)	0.84 (0.59, 1.20)	0.80 (0.59, 1.08)	0.80 (0.58, 1.11)
40– <50 years	1.17 (0.74, 1.85)	1.70 (1.18, 2.45)	1.66 (1.13, 2.44)	1.12 (0.80, 1.58)	1.29 (0.97, 1.72)	1.05 (0.77, 1.45)
50– <60 years	2.50 (1.65, 3.78)	2.24 (1.57, 3.20)	2.54 (1.77, 3.66)	1.55 (1.12, 2.15)	2.14 (1.64, 2.78)	2.12 (1.59, 2.83)
Over 60 years	4.83 (3.26, 7.15)	5.46 (3.94, 7.58)	4.69 (3.31, 6.64)	2.85 (2.11, 3.85)	3.54 (2.74, 4.58)	3.97 (3.01, 5.24)
Male	2.10 (1.70, 2.60)	1.65 (1.38, 1.97)	2.04 (1.70, 2.45)	1.85 (1.54, 2.21)	1.98 (1.71, 2.28)	2.01 (1.72, 2.35)
Living in Hanoi	1.52 (1.23, 1.87)	1.13 (0.94, 1.35)	1.13 (0.94, 1.36)	1.14 (0.95, 1.36)	1.10 (0.95, 1.27)	1.17 (1.00, 1.37)

*Presented in each cell is the proportion ratio and 95% confidence interval (PR and 95%CI)*.

After adjusting for age, sex, COVID-19 risk of the residential area, and GI emergency endoscopy, the proportion of PUD was not different between 2020 and 2019, except a lower proportion in June 2020 compared to June 2019 (Tables 3A,B).

**Table 3:** The log-binomial models for the proportion of PUD.

**Factor**	**January**	**February**	**March**	**April**	**May**	**June**
**A**						
Year 2020	0.88 (0.80, 0.96)	0.97 (0.88, 1.07)	1.00 (0.91, 1.10)	1.12 (0.99, 1.26)	1.04 (0.97, 1.12)	0.89 (0.82, 0.96)
**Age group (reference group: 18–** ** <30 years)**						
<18 years	1.30 (1.02, 1.65)	1.93 (1.49, 2.50)	1.51 (1.19, 1.92)	1.82 (1.38, 2.40)	1.43 (1.16, 1.76)	1.22 (0.99, 1.52)
30– <40 years	1.31 (1.12, 1.53)	1.41 (1.18, 1.68)	1.21 (1.04, 1.42)	1.10 (0.91, 1.34)	1.06 (0.93, 1.21)	1.25 (1.09, 1.44)
40– <50 years	1.23 (1.04, 1.45)	1.62 (1.36, 1.94)	1.35 (1.15, 1.58)	1.42 (1.17, 1.71)	1.19 (1.04, 1.36)	1.35 (1.17, 1.56)
50– <60 years	1.53 (1.30, 1.79)	1.81 (1.52, 2.16)	1.61 (1.38, 1.88)	1.50 (1.24, 1.81)	1.27 (1.11, 1.44)	1.48 (1.28, 1.70)
Over 60 years	1.52 (1.29, 1.79)	1.97 (1.64, 2.37)	1.69 (1.44, 1.99)	1.62 (1.34, 1.97)	1.48 (1.29, 1.70)	1.69 (1.46, 1.96)
Male	1.50 (1.37, 1.64)	1.51 (1.37, 1.66)	1.65 (1.52, 1.80)	1.53 (1.38, 1.70)	1.68 (1.56, 1.81)	1.72 (1.59, 1.86)
Emergency endoscopy	1.42 (1.17, 1.71)	1.19 (0.98, 1.45)	1.29 (1.08, 1.53)	1.35 (1.12, 1.62)	1.26 (1.08, 1.46)	1.36 (1.17, 1.59)
**COVID-19 risk of residential area (reference group: high)**						
Low	0.92 (0.84, 1.02)	0.98 (0.88, 1.10)	0.98 (0.89, 1.07)	1.00 (0.89, 1.12)	0.96 (0.88, 1.05)	1.04 (0.96, 1.14)
Moderate	0.90 (0.79, 1.02)	0.97 (0.85, 1.11)	0.91 (0.81, 1.02)	1.03 (0.89, 1.18)	0.95 (0.85, 1.05)	0.96 (0.87, 1.07)
**B**						
Year 2020	0.87 (0.80, 0.96)	0.97 (0.88, 1.06)	1.00 (0.91, 1.10)	1.09 (0.97, 1.24)	1.04 (0.96, 1.12)	0.89 (0.82, 0.96)
**Age group (reference group: 18–** ** <30 years)**						
<18 years	1.30 (1.02, 1.65)	1.95 (1.51, 2.52)	1.51 (1.19, 1.93)	1.83 (1.39, 2.40)	1.43 (1.16, 1.76)	1.23 (0.99, 1.52)
30– <40 years	1.31 (1.12, 1.53)	1.42 (1.19, 1.70)	1.22 (1.04, 1.43)	1.11 (0.91, 1.35)	1.07 (0.94, 1.22)	1.26 (1.09, 1.45)
40– <50 years	1.23 (1.05, 1.45)	1.64 (1.37, 1.97)	1.37 (1.17, 1.60)	1.44 (1.19, 1.74)	1.20 (1.05, 1.37)	1.36 (1.18, 1.57)
50– <60 years	1.54 (1.31, 1.80)	1.85 (1.55, 2.21)	1.63 (1.40, 1.91)	1.53 (1.26, 1.85)	1.27 (1.11, 1.45)	1.48 (1.28, 1.71)
Over 60 years	1.52 (1.29, 1.80)	2.00 (1.66, 2.41)	1.70 (1.45, 2.00)	1.65 (1.36, 2.00)	1.49 (1.30, 1.71)	1.70 (1.46, 1.97)
Male	1.50 (1.37, 1.64)	1.51 (1.37, 1.67)	1.65 (1.52, 1.80)	1.54 (1.39, 1.71)	1.68 (1.56, 1.82)	1.72 (1.59, 1.86)
Emergency endoscopy	1.41 (1.17, 1.70)	1.19 (0.98, 1.45)	1.28 (1.08, 1.52)	1.35 (1.12, 1.62)	1.25 (1.08, 1.46)	1.37 (1.18, 1.60)
Living in Hanoi	1.10 (1.01, 1.20)	1.13 (1.02, 1.25)	1.09 (1.00, 1.19)	1.12 (1.00, 1.24)	1.08 (1.00, 1.17)	1.02 (0.94, 1.11)

*Presented in each cell is the proportion ratio and 95% confidence interval (PR and 95% CI)*.

Age ≥50 years and male sex were consistently associated with a higher proportion of GI emergency endoscopy and PUD. The GI emergency endoscopy was associated with a higher proportion of PUD. There was no difference in the proportion of GI emergency endoscopy and PUD in low- and moderate-risk areas compared to high-risk areas.

## Discussion

We observed the proportion of both GI emergency endoscopy and PUD was significantly higher during the time of the state of emergency due to the ongoing COVID-19 pandemic in 2020 when compared to 2019 at the same health facility in Vietnam. Our findings suggest that healthcare delivery reforms during the era of an emerging pandemic time are highly needed to reduce digestive disorders, in particular, and chronic diseases in general.

It is apparent that, after the first COVID-19 cases in Vietnam were reported, the number of patients with upper GI endoscopy dropped remarkably. The most significant drop was in early April, which was the period of nationwide distancing in Vietnam, reflecting the commitment of the Vietnamese people and hospitals in complying with the regulations of the country with respect to COVID-19. This situation was similar in other countries during the ongoing COVID-19 pandemic, where endoscopic procedures decreased in volume and were mostly limited to urgent procedures (16–22). Studies have shown that the disruption caused by the COVID-19 pandemic to the GI endoscopy units could delay cancer diagnosis, upshift the cancer stage at diagnosis, and even result in more deaths (3, 23).

Despite the reduction in absolute number, the proportion of GI emergency endoscopy in April 2020 was higher compared to the same month in 2019, even after controlling for age, sex, and COVID-19 risk. The proportion of PUD was also higher in April 2020, although this difference became insignificant after controlling for covariates. Another study reported a similar scenario where the likelihood of diagnosing colorectal cancer increased despite a lower number of cancers detected (17). This might be explained by several elements. Firstly, COVID-19 might have negative impacts on patients in multiple ways, which increased the risk of PUD and its complications. The patients might suffer from stress due to anxiety, economic crisis and unemployment, and long-term space confinement. While staying at home, they are more likely to engage in more harmful behaviors such as alcohol consumption and unhealthy eating (24–27). These have been reported as risk factors in PUD (28, 29). Secondly, during the nationwide distancing, owing to travel restriction, people only visited the hospital when they felt unbearably ill. Therefore, the proportion of outpatients admitted with more severe clinical presentations likely increased for this reason, and so was the proportion of GI emergency endoscopy and PUD. Based on this logic, one might argue that the patients coming from low- and moderate-risk areas should have a higher proportion of GI emergency endoscopy and PUD. However, our findings do not support this argument: There was no difference in the proportion of low- and moderate-risk areas compared to high-risk areas. In general, studies to explore and explain the impact of COVID-19 on the behaviors and health of the patients are urgently needed.

Endoscopists express their concerns about patient subpopulation priority during the COVID-19 pandemic. In April 2020, the A-PSDE released some recommendations on GI endoscopy (2). In addition to instructions on personal protective equipment, the A-PSDE stated that upper GI endoscopy should only be performed in patients who had symptoms suggesting a GI emergency condition or needed upper GI endoscopy for diagnostic confirmation. In areas where SARS-CoV-2 transmission is common, these instructions help limit the risk of transmission in healthcare facilities and allow more time for the hospitals to prepare for personal protective equipment for the healthcare providers. However, in areas with lower COVID-19 incidence, the question is whether this recommendation is too restrictive. Our study shows that the proportion of PUD in both emergency and non-emergency patients increased in April 2020. This means, if we only perform upper GI endoscopy on emergency patients, we will miss more PUD cases in the non-emergency groups than usual. Therefore, in an area where the COVID-19 situation is relatively well-controlled, the hospital might consider upper GI endoscopy for both emergency and non-emergency patients as long as other precautions, such as hand hygiene, use of personal protective equipment, and physical distancing, are guaranteed. If upper GI endoscopy were to be limited in a subpopulation of non-emergency patients, the patients with a higher risk of PUD could be prioritized. In our study, a higher proportion of PUD was associated with male patients and patients aged ≥45 years, which suggests these subpopulations could be a primary target of prioritization. Literature reviews and more extensive studies should be performed to explore other plausible factors that might help triage and prioritize patients.

Our study has certain strengths. We were able to collect the entire dataset within the first 6 months of 2019 and 2020 with very few missing data. The large number of patient intake at HMUH allowed well-stratified and multivariable comparisons. The results that we provide can be used to understand how the practice of upper GI endoscopy is impacted by the COVID-19 pandemic in the context of a low-incidence region. These data can be utilized in health policy analysis studies and can suggest options for countries and hospitals that have a similar context to Vietnam and HMUH.

However, there are important limitations of our study. Firstly, we could not collect the data from other years for serial analyses. Even if we were able to do so, there would be multiple factors that affect the number of patients, such as a change in infrastructure and human resources, which can increase or decrease the capacity of a hospital. Secondly, because our data were collected from the electronic database of the hospital, many data are in free-text format and cannot be extracted, such as a clinical presentation. Therefore, the covariates included in our study were restricted to more structured or extractable data fields.

In conclusion, during the nationwide distancing in April 2020 in Vietnam, the proportion of gastrointestinal emergency endoscopy and peptic ulcer disease was higher compared to April 2019. Male patients and patients aged ≥50 years were associated with a higher proportion of peptic ulcer disease and emergency endoscopy, suggesting these subpopulations should be prioritized if endoscopy were to be limited.

## Data Availability Statement

The datasets presented in this study can be found in online repositories. The names of the repository/repositories and accession number(s) can be found at: https://doi.org/10.7910/DVN/RMFFHJ, Harvard Dataverse, V2, UNF:6:EsFtMlgLOu5L8eTVWy3bCg= [fileUNF].

## Ethics Statement

The studies involving human participants were reviewed and approved by the Institutional Review Board of Hanoi Medical University, 1, Ton That Tung Street, Dong Da district, Hanoi, Vietnam. Written informed consent from the participants' legal guardian/next of kin was not required to participate in this study in accordance with the national legislation and the institutional requirements.

## Author Contributions

HD, LH, and LD conceived the concept and project and design of the study. LD and HD supervised the project. HD, LH, TT, and HN created the model and collected data. LH analyzed the results. HD, LH, and TT wrote the draft manuscript. HD, NNHL, and NTL reviewed and edited the final manuscript. All authors read, edited, and approved the final manuscript.

## Conflict of Interest

The authors declare that the research was conducted in the absence of any commercial or financial relationships that could be construed as a potential conflict of interest.

## Publisher's Note

All claims expressed in this article are solely those of the authors and do not necessarily represent those of their affiliated organizations, or those of the publisher, the editors and the reviewers. Any product that may be evaluated in this article, or claim that may be made by its manufacturer, is not guaranteed or endorsed by the publisher.
